# Incidence management system of the healthcare institutions for disaster management in Sri Lanka

**DOI:** 10.1186/s12873-023-00777-y

**Published:** 2023-01-23

**Authors:** Nayani Umesha Rajapaksha, Chrishantha Abeysena, Aindralal Balasuriya, Millawage Supun Dilara Wijesinghe, Suranga Manilgama, Yibeltal Assefa Alemu

**Affiliations:** 1 Ministry of Health, Nutrition and Indigenous Medicine, Colombo, Sri Lanka; 2 Department of Community Medicine, Faculty of Medicine, Ragama, University of Kelaniya, Kelaniya, Sri Lanka; 3 Department of Public Health, General Sir John Kotelawala Defence University, Ratmalana, Sri Lanka; 4 School of Public Health, Faculty of Medicine, The University of Queensland, Brisbane, Australia

**Keywords:** Incident management system, Incidence command system, Disaster, Management, Healthcare institutions, Surge capacity

## Abstract

**Background:**

Incident management systems and disaster planning processes facilitate maximal use of available resources. Evaluation of the Incident Command System (ICS) is one of the top five key areas of research priority in the field of surge. The study was aimed at assessing the disaster preparedness and ICS of the public healthcare institutions for the disaster management in a disaster-prone district of Sri Lanka.

**Methods:**

A descriptive cross-sectional study was conducted among all public sector healthcare institutions (*n* = 74), including curative-healthcare institutions (*n* = 46) which have inward-care facilities for patient care and preventive healthcare institutions (*n* = 28) in Kurunegala district, Sri Lanka from May–September 2019 using a validated interviewer administered questionnaire which was based on ‘CO-S-TR Model’ for ICS assessment including ‘Clear need for increased capacity (≤25%), Basic level (26 – 50%), Moderate level (51 – 75%) and High level (>75%)’.

**Results:**

Focal points for disaster management were nominated by the majority of the curative sector (*n* = 33; 76.7%) and preventive sector (*n* = 19; 73.1%) healthcare institutions. A written disaster preparedness and response plans were available in 72% (*n*= 31) curative sector and 76% (*n*= 19) preventive sector institutions. The higher proportion of the curative sector institutions had moderate level capacity in the area of providing treatment, and basic level capacities were in the areas of ‘staff mobilization, coordination of activities, supplying of special needs, triage of cases and transportation’. There is a clear need for improvement in the areas of communication commanding, management of controlling the incidence and tracking of the cases in the curative sector. The majority of the preventive sector institutions had moderate level capacity in commanding, control, coordination and tracking of cases. The basic level capacity in the areas of staff mobilization, stuff management and triage of cases. There is a clear need for improvement in the areas of communication in preventive sector. Of the public sector healthcare institutions, the higher proportion of the preventive sector (*n* = 20; 76.9%) and curative sector (*n* = 29; 67.4%) had basic level overall surge capacity of ICS for disaster management.

**Conclusion:**

Coordination, communication, commanding, management of controlling the incidence and tracking of cases following outbreaks need to be improved and capacity development programmes could implement to develop the preparedness for future disasters.

**Supplementary Information:**

The online version contains supplementary material available at 10.1186/s12873-023-00777-y.

## Background

Disaster is defined as “a serious disruption in the functioning of a community or society involving widespread human, material, economic, or environmental losses and impacts that exceed the affected community’s or society’s ability to cope using its own resources” [1]. Disaster management is a cyclical process with three key elements including preparedness, response, and recovery. During the preparedness phase, measures should be implemented in order to ensure the coordinated planning and implementation for effective relief. A set of activities executed following the impact of a disaster is included in the response phase. During the response phase, measures to assess needs and minimize suffering and disaster consequences should be implemented. The rehabilitation and reconstruction phases comprise the recovery phase. During the rehabilitation phase, the restoration of basic social functions is ensured [2]. Surge capacity describes the ability of a healthcare system to respond to a sudden increase in patient care demands [3]. However, there are three key dimensions of surge capacity. The dimensions of surge capacity include, ‘healthcare facility-based surge capacity, public health surge capacity, and community-based surge capacity’ [4]. Surge capacity consists of four major key components. Structure for disaster management is one of the key components in assessing surge capacity [5] and structure refers to facilities and specific organizational structures such as Incident Command System (ICS) [6–8]. Surge capacity of ICS is defined as the ability to manage a sudden unexpected increase in patient volume that would otherwise severely challenge or exceed the capacity of the current healthcare system [8]. Incident management systems and cooperative planning processes facilitate maximal use of available resources [4]. Moreover, the initial command goals are to protect the staff, facility and prevent event expansion [3]. Evaluation of the ICS is one of the top five key areas of research priority in the field of surge [9]. The ICS is a model for command, control, and coordination of emergency response at the site level. The same ICS structure can also be used to coordinate site support at regional, provincial or national level emergency operation centers [10]. Implementation of the essential elements of the ICS using ‘CO-S-TR model’ should include a brief, facility specific mobilization checklist to enable rapid identification and prioritization of resource needs, recognition of key objectives, and earlier incident control [3].

In terms of occurrence (44%), fatalities (72%), and victims (60%), Asia was the most affected continent by natural disasters. Droughts, floods, extreme temperatures causing heat waves, and the El Nino effect constituted the most common [11]. Sri Lanka is an Asian country that is vulnerable to a variety of hazards that can result in disasters. The vulnerable population must suffer as a result of disasters. Droughts, floods, landslides, cyclones, vector-borne epidemics, and coastal erosion are the most common natural hazards in Sri Lanka. Tsunamis are uncommon, but the 2004 Tsunami caused extensive damage primarily in the coastal areas of the Northern, Eastern, and Southern provinces. The Indian Ocean region’s tectonics also pose an increasing risk of earthquakes [12]. With improved infrastructure, such as road widening and highway construction, there is an increased risk and frequency of transportation accidents. Political instability in a country can increase the likelihood of strife, protests, and civil conflicts. According to the Disaster Preparedness and Response Division (DPRD) of Sri Lanka, the primary concern of any disaster is to minimize human suffering, which is where the health sector comes in. As a result, it is critical that the health sector is prepared to respond effectively and efficiently in the event of a disaster. According to the National Strategic Plan for the Health Sector in Sri Lanka, the Disaster Management Act No. 13 of May 2005 was enacted to develop the country’s capacity for disaster management with the goal of ensuring the least amount of human suffering. The legal provision stated that preparing the health sector for effective and efficient response in accordance with the disaster management act is the most important strategy for building health sector capacity. To overcome the temporary mismatch between demand and supply of care following disasters, institutional capacity should be developed during the preparedness phase. Physical infrastructure, resources, organizational structure, expertise, skills, training, and attitudes of staff, as well as other emergency response capabilities, are all examples of capacity. Health managers must conduct a capacity assessment in order to identify the activities that must be completed on a priority basis [13]. Effective disaster management should be consistent with disaster preparedness and response planning. Disease-endemic and disaster vulnerable countries, however, neglect it. The measures should be adapted to the countries’ specific circumstances. Capacity assessments are more important than encouraging stakeholders to participate in response management. To achieve sustainable development in institutions, a systematic approach to capacity development programme planning is required. Before developing long-term capacity development strategies, capacity assessment surveys should be conducted to detect existing needs and demand for capacity development. However, identifying the items, personnel, systems, and structures that comprise the healthcare delivery system is a difficult task [14–16]. As a result, there was a need to conduct a systematic assessment of healthcare institutions’ surge capacity including ICS. The aim of this study was to assess the disaster preparedness and ICS of the public healthcare institutions for disaster management in a disaster-prone major district of Sri Lanka.

## Methods

A descriptive cross-sectional study was conducted among all healthcare institutions (*n* = 74) including curative-healthcare institutions (*n* = 46) which have inward-care facilities for patient care and preventive healthcare institutions (*n* = 28) in Kurunegala District of Sri Lanka’s Northwestern Province (NWP) from May–September 2019. Sri Lanka has a land area of 65,000 km^2^ and is divided into nine administrative provinces, according to the Census and Statistics Department. The NWP is one of nine provinces and is comprised of two districts: Kurunegala and Puttalam. The country’s total population was reported to be 20,359,439. NWP housed 12% (*n* = 2,380,861) of the Sri Lankan population. The NWP had a total population of 2,380,861 people. The Kurunegala district had the highest population density of 350 people per square kilometers (68%; *n* = 1,618,465) of NWP [17]. Recent climate change-related events and improper land use are also influencing current disaster patterns. Animal attacks are another common tragedy. Within the 17 districts, wild elephant habitats cover 33% of the land. Conflicts between humans and elephants have been reported in wildlife areas, including NWP. Elephant attacks claimed the lives of 11,341 people between 2008 and 2012. In 2012 alone, 79 people were killed, with the highest numbers reported from the NWP, Northern, and Eastern Provinces [18]. When it comes to disease outbreaks, Dengue fever is becoming more common and severe around the world, and it has become a major public health issue in Sri Lanka. Dengue fever has been on the rise in Sri Lanka for the past two decades. During the most recent major epidemic in Sri Lanka, disease outbreaks severely affected 15 of the 26 districts. Kurunegala experienced a significant increase in dengue cases in 2017. There is evidence that the Kurunegala district has experienced annual outbreaks for the last two decades [12]. Notably, the management capacity of the Kurunegala district’s government healthcare institutions was exceeded due to the disease outbreaks including dengue and COVID-19, resulting in a disaster for the district in the recent outbreaks. The interviewer-administered questionnaire (IAQ) was based on ‘CO-S-TR Model’ and data was collected from an authorized person for disaster management in each institution. There are three major categories in the tool, each with ‘four sub elements including: “CO” - command, control, communications, and coordination which ensures that an incident management structure is implemented; “S” considers the logistical requirements for staff, stuff, space, and special considerations; “TR” comprises tracking, triage, treatment, and transportation for basic patient care and patient movement functions’ [3]. Therefore, the CO-S-TR Model consists of 12 areas of the surge capacity assessment which is intended as an adjunct tool to the ICS and similar systems. The drafted IAQ was validated (face, content, and consensual validity-judgement validity) to improve the efficiency of the study through the guidance of a panel of experts using the Delphi technique. The use of the Delphi technique instead of face-to-face consultative meetings had the advantage of not requiring the experts to take time off their schedules to contribute to the study. It allowed the experts to respond at any time convenient to them and to contact any source of information if needed. Further, this process facilitated the independence of forming opinion and perspectives as it prevented the manipulation of opinion by influential individuals, which could happen in a face-to-face consultative meeting [19]. The CO-S-TR model has originally been developed as a self-administered checklist which is intended as an adjunct tool to the hospital ICS and similar systems. However, the originators have offered the option of using it as an IAQ to have better results. Two data collectors were used to minimize inter-observer variation. Data collectors were trained specifically on measures to ensure uniform administering of the tool. At the end of the training session, the data collectors were given the opportunity to conduct mock interviews and those interviews were observed and feedback on improving the interviewing techniques were provided to improve the quality. The mean completion time of the basic evaluation of the tool was found to be approximately 30 minutes which is shorter than in many other surge capacity assessment tools. It is a feature which is considered important to ensure compliance of the respondents and to generate quality data. Data analysis was done using SPSS version 22. Equal mark of 25 was given to each sub-element of the CO-S-TR tool and total percentage was taken for each institution. The range was zero to 100% for the total elements of the tool. An aggregated percentage mark of each category was analyzed and described according to the following ranking system considering four levels of capacity components including ‘Clear need for increased capacity (≤25%), Basic level of capacity in place (16–50%), Moderate level of capacity in place (51–75%) and High level of capacity in place (> 75%)' [Refer additional file 1].

## Results

The results describe the curative and preventive healthcare institutions in the district separately.

### System for disaster management

Focal points for disaster management were nominated by the majority (*n* = 33; 76.7%) curative sector healthcare institutions. Of them, higher proportion (*n* = 21; 63.6%) had working experience more than 10 years in the health sector and 45.5% (*n* = 15) of them has been working in the current healthcare institution for more than 3 years. Out of the 33 focal points, 42.4% (*n* = 14) had experience in managing medical disasters, 78.8% (*n* = 26) had experience in managing surgical disasters and 36.4% (*n* = 12) had experience in managing both types of disasters. Only 27.3% (*n* = 9) had undergone any formal disaster management training during their lifetime. Focal points for disaster management were nominated by the majority (*n* = 19; 73.1%) of the preventive sector healthcare institutions. Of them, 63.2% (n = 12) had working experience more than 10 years in the health sector and 52.6% (*n* = 10) of them has been working in the current health care institution for more than 3 years. Out of the 19 focal points, 68.4% (*n* = 13) had experience in managing medical disasters, 36.8% (*n* = 7) had experience in managing surgical disasters and 31.6% (*n* = 6) had experience in managing both medical and surgical disasters. Only 26.3% (*n* = 5) had undergone disaster management training during last 15 years (Table 1).

**Table 1:** Availability of Focal Points for Disaster Management in the Institution and their Working Experience, Experience in Managing Disasters and Formal Training received in Curative Sector and Preventive Sector Healthcare Institutions

	Category	Healthcare Institution	
		Curative sector	Preventive sector
		n (%)	n (%)
**Focal point**	Not nominated	10 (23.3%)	7 (26.9%)
	Nominated	33 (76.7%)	19 (73.1%)
	**Total**	**43 (100.0%)**	**26 (100.0%)**
**Working experience of the focal point**	10 years or less	12 (36.4%)	7 (36.8%)
	More than 10 years	21 (63.6%)	12 (63.2)
	**Total**	**33 (100.0%)**	**19 (100.0%)**
**Experiences of the focal points**	3 years or less	18 (54.5%)	10 (52.6%)
	More than 3 years	15 (45.5%)	9 (47.4%)
**Experience in disaster management**	**Medical disasters**		
	No experience	19 (57.6%)	6 (31.6%)
	Experienced	14 (42.4%)	13 (68.4%)
	**Surgical disasters**		
	No experience	7 (21.2%)	12 (63.2%)
	Experienced	26 (78.8%)	7 (36.8%)
	**Both Medical and surgical disasters**		
	No experience	21 (63.6%)	13 (68.4%)
	Experienced	12 (36.4%)	6 (31.6%)
**Training**	Not received a formal training	24 (72.7%)	14 (73.7%)
	Received a formal training	9 (27.3%)	5 (26.3%)
	**Total**	**33 (100%)**	**19 (100.0%)**

### Preparedness and response planning for disaster management in both curative and preventive sector healthcare institutions

Of all responded curative sector institutions (*n* = 43), 23.3% (*n* = 10) did not start to plan for disaster preparedness and response activities. There were 72.1% (*n* = 31) written disaster preparedness and response plans. Of the one who had written plans (n = 31), 51.6% (*n* = 16) has updated on 2019, only 6.5% (*n* = 2) completed the medical disaster management such as dengue, pandemic influenza etc. and 29.0% (*n* = 9) completed the surgical disaster preparedness and response such as mass casualty incidents following bomb blasts, road traffic accidents etc. Of them, 21.2% (*n* = 7) conducted evaluation of the plan and 33.3% (*n* = 11) planned to conduct evaluation of the written plan. Out of the 25 preventive sector institution, 76.0% (*n* = 19) did not have written plan for disaster preparedness and response. When considering the content of the plan who had and in the process of planning, only 8% (*n* = 2) completed the medical disaster management and 32% (*n* = 8) completed the surgical disaster preparedness and response. No one conducted evaluation of the plan and 36.0% (*n* = 9) planned to conduct evaluation of the written plan (Table 2).

**Table 2:** Preparedness and Response planning for Disaster management in both Curative and Preventive Sector Healthcare Institutions

Disaster preparedness and response	Healthcare Institution	
	Curative Sector	Preventive Sector
**Written plan**	**n (%)**	**n (%)**
Not started	10 (23.3%)	6 (24.0%)
In progress	18 (41.9%)	13 (52.0%)
Completed	15 (34.9%)	6 (24.0%)
**Total**	**43 (100.0%)**	**25 (100%)**
**Content of the plan**		
**Medical disasters**		
Not yet started	2 (6.5%)	3 (12.0%)
In progress	1 (3.2%)	20 (80.0%)
Completed	28 (90.3%)	2 (8.0%)
**Total**	**31 (100.0%)**	**25 (100.0%)**
**Surgical disasters**		
Not yet started	14 (45.2%)	3 (12.0%)
In progress	1 (3.2%)	14 (56.0%)
Completed [Last edition in 2019]	16 (51.6%)	8 (32.0%)
**Total**	**31 (100.0%)**	**25 (100.0%)**
**Evaluation of the plan**		**n (%)**
Not yet planned	14 (45.2%)	16 (64.0%)
Plan to conduct	16 (51.6%)	9 (36.0%)
Conducted [Last drill in 2018]	1 (3.2%)	0 (0.0%)
**Total**	**31 (100.0%)**	**25 (100.0%)**

Non-respondent of preventive sector = 1

### The ICS for disaster Management at Curative Sector Institutions

The higher proportion of the curative sector institutions had moderate level capacity in the area of providing treatment (60.5%; *n* = 26). The higher proportion of the institutions had basic level capacity in the areas of staff mobilization (44.2%; *n* = 19), coordination of activities (40.5%; *n* = 20), supplying of special needs (60.5%; n = 26), triage of cases (48.8%; *n* = 21) and transportation (55.8%; *n* = 24). There is a clear need for improvement in the areas of communication (81.4%; *n* = 35), commanding (62.8%; *n* = 27), management of controlling the incidence (53.5%; *n* = 23) and tracking of the cases following disease outbreaks (46.5%; *n* = 20) (Table 3).

**Table 3:** The ICS for Disaster Management at Curative Sector Institutions

	Action area	Levels of capacity of ICS			
		Clear need	Basic level	Moderate	High level
**1**	**Commanding**	**27 (62.8%)**	9 (20.9%)	6 (14.0%)	1 (2.3%)
**2**	**Control**	**23 (53.5%)**	13 (30.2%)	6 (14.0%)	1 (2.3%)
**3**	**Communication**	**35 (81.4%)**	5 (11.6%)	3 (7.0%)	0 (0.0%)
**4**	**Coordination**	17 (39.5%)	**20 (46.5%)**	6 (14.0%)	0 (0.0%)
**5**	**Staff mobilization**	15 (34.9%)	**19 (44.2%)**	9 (20.9%)	0 (0.0%)
**6**	**Stuff**	**33 (76.7%)**	8 (18.6%)	2 (4.7%)	0 (0.0%)
**7**	**Space**	17 (39.5%)	**20 (46.5%)**	5 (11.6%)	1 (2.3%)
**8**	**Special needs**	1 (2.3%)	**26 (60.5%)**	15 (34.9%)	1 (2.3%)
**9**	**Tracking of cases**	**20 (46.5%)**	18 (41.9%)	4 (9.3%)	1 (2.3%)
**10**	**Triage of cases**	10 (23.3%)	**21 (48.8%)**	12 (27.9%)	0 (0.0%)
**11**	**Treatment**	6 (14.0%)	10 (23.3%)	**26 (60.5%)**	1 (2.3%)
**12**	**Transportation**	9 (20.9%)	**24 (55.8%)**	10 (23.3%)	0 (0.0%)

### The ICS for disaster Management at Preventive Sector Institutions

The majority of the preventive sector healthcare institutions had moderate level capacity in commanding (*n* = 11; 42.3%), control (*n* = 10; 38.5%), coordination (*n* = 16; 61.5%) and tracking of cases (*n* = 20; 76.9%). The basic level capacity in the areas of staff mobilization (*n* = 14; 53.8%), stuff management (n = 20; 76.9%), Space (16; 61.5%) and triage of cases (n = 20; 76.9%). There is a clear need for improvement in the areas of communication (*n* = 12; 46.2%) among the higher proportion (Table 4).

**Table 4:** The ICS for Disaster Management at Preventive Sector Institutions

	Action area	Levels of capacity of ICS			
		Clear need for improvement	Basic level	Moderate level	High level
**1**	**Commanding**	3 (11.5%)	4 (15.4%)	**11 (42.3%)**	8 (30.8%)
**2**	**Control**	1 (3.8%)	6 (23.1%)	**10 (38.5%)**	9 (34.6%)
**3**	**Communication**	**12 (46.2%)**	6 (23.1%)	7 (26.9%)	1 (3.8%)
**4**	**Coordination**	0 (0.0%)	6 (23.1%)	**16 (61.5%)**	4 (15.4%)
**5**	**Staff mobilization**	1 (3.8%)	**14 (53.8%)**	11 (42.3%)	0 (0.0%)
**6**	**Stuff**	1 (3.8%)	**20 (76.9%)**	5 (19.2%)	0 (0.0%)
**7**	**Space**	0 (0.0%)	**16 (61.5%)**	6 (23.1%)	4 (15.4%)
**8**	**Special needs**	**7 (26.9%)**	6 (23.1%)	13 (50.0%)	0 (0.0%)
**9**	**Tracking of cases**	0 (0.0%)	4 (15.4%)	**20 (76.9%)**	2 (7.7%)
**10**	**Triage of cases**	0 (0.0%)	**20 (76.9%)**	6 (23.1%)	0 (0.0%)
**11**	**Transportation**	3 (11.5%)	**12 (46.2%)**	11 (42.3%)	0 (0.0%)

*Treatment services are not provided during disasters by the preventive sector

### Overall surge capacity of the ICS of the public sector healthcare institutions for the Management of Disasters

There is one Provincial General Hospitals (PGH) in Kurunegala district which has moderate level overall capacity of the ICS for disaster management. Out of the four Base Hospitals (BH), higher proportion (75%; *n* = 3) had moderate level surge capacity and out of 38 District Hospitals (DH), higher proportion (76.3%; *n* = 29) had basic level capacity of the ICS for disaster management. Of the 26 public sector preventive healthcare institutions, the higher proportion (76.9%; *n* = 20) had basic level surge capacity for disaster management (Table 5).

**Table 5:** Overall Surge Capacity of the ICS of the Public Sector Healthcare Institutions for the Management of Disaster

Type of institutions	Levels of Overall Surge Capacity of the ICS				Total
	Clear need for improvement	Basic level	Moderatelevel	High Level	
**Preventive sector healthcare institutions**					
**MOH**	**2 (7.7%)**	**20 (76.9%)**	**4 (15.4%)**	**0 (0.0%)**	**26 (100.0%)**
**Curative sector healthcare institutions**					
**PGH**	0 (0.0%)	0 (0.0%)	1 (100.0%)	0 (0.0%)	1 (100.0%)
**BH**	0 (0.0%)	0 (0.0%)	3 (75.0%)	1 (25.0%)	4 (100.0%)
**DH**	1 (2.6%)	29 (76.3%)	8 (21.1%)	0 (0.0%)	38 (100.0%)
**Total**	**1 (2.3%)**	**29 (67.4%)**	**12 (27.9%)**	**1 (2.3%)**	**43 (100.0%)**
**Overall**	**3 (4.3%)**	**49 (71.0%)**	**16 (23.2%)**	**1 (1.4%)**	**69 (99.9%)**

*MOH = Medical Officers of Health areas, PGH = Provincial General Hospitals, BH = Base Hospitals, DH = District Hospitals

## Discussion and limitations

Incident command system (ICS) is a specific organizational structure for emergency management, which support the effective management of surge capacity [20]. However, due to unpredictable nature of the disasters, management according to available plans and structures may be difficult [21, 22]. The ICS provides guidance for response with available resources following an incident and operationalization of the activities. All response activities can be categorized in to five functional areas including ‘command, operations, planning, logistics, and administration’. The core concepts of the ICS are ‘common terminology; integrated communications; modular organization; assets within each functional unit may be expanded or contracted based on the requirements of the event, unified command structure, manageable span of control, consolidated action plans; comprehensive resource management and pre-designated incident facilities’ [23]. Importantly, surge capacity of the Emergency Department (ED) and hospitals which provide inward care has been studied by Kanter and Moran. It is difficult to plan disaster response until assessing disaster need and existing hospital resources. All type of healthcare institutions in all settings face challenges to meet surge demands during massive influx of cases [24]. According to a study on daily ED surge capacity, systems, space, staffing, and supplies are the multiple dimensions of the ED surge capacity and there are many factors influencing. Because of unplanned emergency services in many hospitals, the EDs face significant surges in demand on a daily basis [25]. The hospitals need to have the capacity to manage critical ill patients at least 10 days without external support following any mass casualty incident [9]. Importantly, there is a need of the research to quantify the impact and effectiveness in the context of surge. All the staff in the institution are responsible for dealing with surge [26]. Further, when considering the effective systems planning, “internal and external communication processes” need to be considered. There should be contingency plans when proper plans go wrong [27]. However, all staff need to be familiar with communication plans and the mechanism of implementation need to be established. Furthermore, alternate communication devices within the facility, such as two-way radios need to be familiar with the whole staff [28]. Otherwise, the purpose of better management will not be happened. According to the study findings, when considering the ICS in the healthcare institutions, the moderate level capacity was existed in the higher proportion of curative sector healthcare institutions in provision of treatment and basic level capacity in the areas including, coordination, staff, space, special, triage and transportation during an emergency. There is a clear need for improvement in the areas of commanding system activation, control of the incidence, communication, stuff and tracking of cases during outbreak and other mass casualty incidents.

A well-defined ICS helps to collect and process information, develop incident plans, and to manage decisions is essential to manage mass casualty incidents [29]. The results of the present study indicated that the higher proportion of the preventive sector institutions had moderate level capacity in the areas of commanding system, controlling the incidence, coordination of activities, supplying of special needs and tracking of the cases. The higher proportion of the curative sector healthcare institutions had basic level capacity in management of the stuff, triage of case, and transportation. There is a clear need for improvement in the areas of activation of command system, control of the incidence, communication during management, management of other stuff and tracking of cases during outbreaks. Further, when considering disaster preparedness, the focal points aim to integrate disaster management and to promote coherence [30]. They usually act as the emergency coordinator for the institution. The coordinator is responsible for technical and administrative duties, and they must be capable of coming up with preparedness plans that will minimize damages and complications in the event of an emergency [31]. According to the Disaster Management Act no.13, 2005, Sri Lanka, every institution should have a focal point for disaster management and disaster preparedness plan for their institution. There are several studies have stated that there is poor response capacity of healthcare workers due to lack of experience in managing disasters [32–34] and the experience in the management of disasters leads to improve the capacity to handle disaster situations [35, 36]. Furthermore, there are several studies have revealed that the conduction of the disaster training programs of the institutions lead to development of institutional capacity [32, 37, 38] and there was a statistically highly significant (*p* < 0.001) associations between the training received on disaster management with the good attitudes of the healthcare workers [16]. Effective systems planning supports development of decision-making capacity. This includes having effective decision-making processes in place, with multiple staff members prepared and empowered to make key decisions as needed [3, 4]. Another element of systems planning is the relationship of the healthcare institution with the surrounding community and region. Administrators should plan to strength the stakeholder partnership and relationships may need to be established within the community [3, 39, 40]. The coordinators should anticipate being involved in negotiations for such relationships. At all levels of a healthcare institutions, healthcare workers will play a role in operationalizing the relationships. Proper planning with the participation of stakeholders in the best way to overcome the challenging task during response activities. Therefore, all the administrators should take planning for disaster as a priority of their duty. They should consider alternate uses of existing facilities of opportunity that can be adapted into surge hospitals [39]. Furthermore, the disaster coordinators should be aware of additional in-network facilities such as sub-acute units and skilled-staff facilities for coordination of activities and supplying of services [6, 10]. Consideration of mobile and portable facilities are important, because of the potential challenges of staffing and supplying other alternative care sites [39]. Sri Lanka is a developing country, which has limited resources. Therefore, proper planning process will help to manage the disaster situations with available resources in an effective manner. It will invariably help to develop the surge capacity of the institution for disaster management. Despite conducting a thorough literature search, the principal investigator was unable to locate research on the ICS assessment in Sri Lankan healthcare settings. There have been few studies on assessing few determinants. All studies highlighted the value of assessing disaster preparedness and ICS [16, 33, 41, 42].

## Conclusion and implications

Coordination, communication, commanding, management of controlling the incidence and tracking of cases following outbreaks need to be improved and capacity development programmes could implement to develop the preparedness for future disasters. Adequate staff capacity and proper way of staff mobilization are priority areas in surge capacity development. The results of this present study indicated that the higher proportion of the public sector healthcare institutions had basic level capacity in the staff mobilization. Staff mobilization for emergency is a challenging task and seeking supplemental supportive staff from other organizations is also very important [43]. Additional support may come from community members and voluntary organizations following disasters [5, 39, 44]. Sharing of staff with the available health care institutions is also a requirement during an emergency [40]. Administrators, coordinators, focal points and team leaders should consider ways to alleviate the barriers to adequate staffing within the institution or stakeholder support that can help alleviate barriers for training and seeking additional staff [40, 45]. Therefore, all the institutions need to assess the system for disaster management of the institutions and plan for emergency situations in an effective way to overcome the future challenges. This study was limited to a major district of 26 districts in Sri Lanka. Moreover, in depth analysis of each component would give a clear picture of their capacity to manage outbreaks.

### Future research directions and opportunities

The preparation of a plan is an essential part of improving the capacity of healthcare institutions, and capacity development programmes must be carried out after assessing the surge capacity of each institution. Disaster plan should be updated, and simulation drills should be performed at regular intervals to improve the level of experiences and awareness of the components of the disaster plan and to achieve targeted goals. The results can guide health care planners in updating plans and developing guidelines. Despite limited evidence, a systematic review on the effectiveness of hospital staff disaster training methods concluded that disaster drills could be effective in training hospital staff on disaster management. According to the available evidence from that comprehensive review, hospital disaster drills effectively allow health care workers to become familiar with disaster procedures, identify problems in incident command, communication, triage, patient flow, materials and resource management, and crowd control [46]. Furthermore, they allow for the application of lessons learned during real-time disaster response. Disaster drills are an effective way to assess the hospital’s preparedness for real-world disasters and provide an opportunity to improve on previous experiences [46, 47]. Impending problems can be identified and addressed sooner if drills are conducted on a regular basis [34].

## Supplementary Information

**Additional file 1.**

## Data Availability

The datasets used and/or analysed during the current study are available from the corresponding author on reasonable request.

## Abbreviations

ICS: Incident Command System
CO-S-TR: “CO” - command, control, communications, and coordination; "S" - staff, stuff, space, and special considerations; “TR” - tracking, triage, treatment, and transportation
IAQ: The interviewer-administered questionnaire
PGH: Provincial General Hospital
BH: Base Hospital
DH: District Hospital
MOH: Medical Officers of Health areas

## References

[1] UNISDR. Terminology. 2009. https://www.unisdr.org/we/inform/terminology.

[2] WHO/ EHA. Disasters and emergencies, definitions-Training package. 2002. http://apps.who.int/disasters/repo/7656.pdf.

[3] Hick JL, Koenig KL, Barbisch D, Bey TA (2008). Surge capacity concepts for health care facilities: the CO-S-TR model for initial incident assessment. Disaster medicine and public health preparedness.

[4] Hick JL, Hanfling D, Burstein JL, DeAtley C, Barbisch D, Bogdan GM, Cantrill S (2004). Health care facility and community strategies for patient care surge capacity. Ann Emerg Med.

[5] Kaji A, Koenig KL, Bey T (2006). Surge capacity for healthcare systems: a conceptual framework. Acad Emerg Med Off J Soc Acad Emerg Med.

[6] Barbisch DF, Koenig KL (2006). Understanding surge capacity: essential elements. Acad Emerg Med.

[7] Phillips S (2006). Current status of surge research. Acad Emerg Med.

[8] Schultz CH, Koenig KL (2006). State of research in high-consequence hospital surge capacity. Acad Emerg Med.

[9] Rothman RE, Hsu EB, Kahn CA, Kelen GD (2006). Research priorities for surge capacity. Acad Emerg Med.

[10] The National Academy of Sciences. Regional Disaster Response Coordination to Support Health Outcomes: Summary of a Workshop Series. Washington (DC), National Academies Press; 2015. 25879125

[11] CRED. Cred Crunch Newsletter, Issue No. 45 December 2016 - preliminary data: Human impact of natural disasters. 2016. http://reliefweb.int/report/world/cred-crunch-newsletter-issue-no-45-december-2016-preliminary-data-human-impact-natural.

[12] Zubair L, Ralapanawe V, Tennakoon U, Yahiya Z, Perera R. Natural disaster risks in Sri Lanka: Mapping Hazards and Risk Hotspots. 2006. http://www.water.columbia.edu/files/2011/11/Zubair2006NaturalDisaster.pdf.

[13] DPRD (2011). Strategic plan for health sector disaster/emergency preparedness.

[14] Davis A, Lemma T. Capacity development: A UNDP primer. 2006. www.undp.org/capacity.

[15] Runge-Ranzinger S, Kroeger A, Olliaro P, McCall PJ, Sánchez Tejeda G, Lloyd LS (2016). Dengue contingency planning: from research to policy and practice. PLoS Negl Trop Dis.

[16] Rajapksha N, Vallipuranadan M, Fernando D (2015). Capacity assessment for the management of mass fatality incidents following disasters in DGH Trincomalee, Sri Lanka, 2014. Journal of Kurunegala Clinical Society (JKCS).

[17] Department of Census & statistics Sri Lanka. Sri Lanka Census of Population and Housing, 2011 http://www.statistics.gov.lk/PopHouSat/CPH2011/index.php?fileName=NWP&gp=Activities&tpl=3.

[18] Ministry of Disaster Management. Sri Lanka comprehensive disaster management program (SLCDMP) (2014). Colombo.

[19] Hsu C, Sandford B (2007). The Delphi technique: making sense of consensus. Pract Assess Res Eval.

[20] Seyedin, Ryan & Keshtgar. Disaster Management Planning for Health Organizations in a Developing Country. 2011. https://ascelibrary.org/doi/full/10.1061/%28ASCE%29UP.1943-5444.0000045.

[21] Fahlgren TL, Drenkard KN (2002). Healthcare system disaster preparedness, part 2: nursing executive role in leadership. The Journal of nursing administration.

[22] Filmer LB, Ranse J (2013). Who is my leader? A case study from a hospital disaster scenario in a less developed country. Australasian emergency nursing journal : AENJ.

[23] Assistant secretary for preparedness and response (ASPR). What is Medical Surge? 2012. https://www.phe.gov/Preparedness/planning/mscc/handbook/chapter1/Pages/whatismedicalsurge.aspx.

[24] Kanter RK, Moran JR (2007). Hospital emergency surge capacity: an empiric New York statewide study. Ann Emerg Med.

[25] McCarthy ML, Aronsky D, Kelen GD (2006). The measurement of daily surge and its relevance to disaster preparedness. Acad Emerg Med.

[26] Jenkins JL, O’Connor RE, Cone DC (2006). Differentiating large-scale surge versus daily surge. Acad Emerg Med.

[27] Cantrill S, Bonnett C, Hanfling D, Pons PA, care sites. In Phillips, S.J., Knebel, A. (2007). Mass medical care with scarce resources: A community planning guide. (AHRQ publication no. 07–0001).

[28] International Nursing Coalition for Mass Casualty Education. Educational competencies for entry-level registered nurses related to mass casualty incidents. 2003. www.nursing/vanderbilt.edu/incmce/competencies.html.

[29] Barbera JA, Macintyre AG. Medical surge capacity and capability:A management System for integrating medical and health resources during large-scale emergencies. 2007 September. https://www.phe.gov/preparedness/planning/mscc/handbook/documents/mscc080626.pdf.

[30] CapaCities. What is a focal point? (2018–2019 ). http://drm-capacities.eu/drm-curriculum/what-is-a-focal-point/#:~:text=The%20term%20%E2%80%9Cfocal%20point%E2%80%9D%20is,less%20commonplace%20in%20DRM%20policies.&text=The%20focal%20points%20aims%20to,for%20internal%20and%20external%20stakeholders.

[31] Healthcare administration degree programs. What is an Emergency Management Coordinator? 2020. https://www.healthcare-administration-degree.net/faq/what-is-an-emergency-management-coordinator/.

[32] Zhiheng Z, Caixia W, Jiaji W, Huajie Y, Chao W, Wannian L (2012). The knowledge, attitude, and behavior about public health emergencies and the response capacity of primary care medical staffs of Guangdong Province, China. BMC Health Services Research.

[33] Kugaendiran VK, experiences and training received on emergency preparedness among doctors in Trincomalee district. (2014). Diploma in health sector disaster management research.

[34] Al-Ali NM, Ibaid AHA (2015). Health-care providers’ perception of knowledge, skills and preparedness for disaster management in primary health-care centers in Jordan. East Mediterr Health J.

[35] Frykberg ER (2004). Principles of mass casualty management following terrorist disasters. Ann Surg.

[36] Otim S. A case-based knowledge management system for disaster management: fundamental concepts. In: Paper presented at the 3rd international ISCRAM conference. Newark, USA; 2006.

[37] Li X, Huang J, Zhang H (2008). An analysis of hospital preparedness capacity for public health emergency in four regions of China: Beijing, Shandong, Guangxi, and Hainan. BMC Public Health.

[38] Glow SDG, Colucci VJ, Allington DR, Noonan CW, Hall EC (2013). Managing multiple-casualty incidents: A rural medical preparedness training assessment. Pre-hospital and disaster medicine.

[39] Cantrill S, Bonnett C, Hanfling D, Pons P, Phillips SJ, Knebel A (2007). Alternative care sites. Mass medical care with scarce resources: A community planning guide.

[40] Rubinson L, Hick JL, Hanfling DG, Devereaux AV, Dichter JR, Christian MD, Talmor D, Medina J, Curtis JR, Geiling JA, Task Force for Mass Critical Care (2008). Definitive care for the critically ill during a disaster: a framework for optimizing critical care surge capacity: from a Task Force for Mass Critical Care summit meeting, January 26-27, 2007, Chicago, IL. Chest.

[41] Sathishka AMDR (2016). Knowledge Attitudes, and Practices of Medical officers on managing and transfering casualty admissions during a disaster at Base Hospital Gampola, in Kandy. postgraduate Institute of Medicine.

[42] Wickrama KWBK (2013). Knowledge attitudes and practices on disaster preparedness among health care workers in PGH Badulla. Postgraduate Institute of Medicine.

[43] Christian MD, Devereaux AV, Dichter JR, Geiling JA, Rubinson L (2008). Definitive care for the critically ill during a disaster: current capabilities and limitations. CHEST.

[44] Koenig HG (2007). Emergency response system in the United States. South Med J.

[45] Qureshi K, Gershon RRM, Sherman MF, Straub T, Gebbie E, McCollum M (2005). Health care workers’ ability and willingness to report to duty during catastrophic disasters. Journal of Urban Health: Bulletin of the New York Academy of Medicine.

[46] Hsu EB, Jenckes MW, Catlett CL, Robinson KA, Feuerstein C, Cosgrove SE, Green GB, Bass EB (2004). Effectiveness of hospital staff mass-casualty incident training methods: A systematic literature review. Pre-hospital and Disaster medicine.

[47] Jan FA, Tabish SA, Shaheen MA, Kumar D (2005). Mass casualty management-our experiences. JK-Practitioner.

