# Supplementary material for: Incidence management system of the healthcare institutions for disaster management in Sri Lanka
Source: BMC Emerg Med. 2023 Jan 23;23:6. doi: 10.1186/s12873-023-00777-y (PMC9867999; doi:10.1186/s12873-023-00777-y)
Supplement: Supplementary file 1 — Additional file 1. [file 12873_2023_777_MOESM1_ESM.docx]

| **Type of the institution** | | | | |
| --- | --- | --- | --- | --- |
| 1. **Preventive sector** |  | | | |
| 1. **Curative sector** | | | | |
| **1. Teaching Hospital (TH)** |  | | | |
| **2. Provincial General Hospital (PGH)** |  | | | |
| **3. Base Hospital** | **BH A** | | **BH B** | |
| **4. Divisional Hospital** | **DH A** | **DH B** | | **DH C** |

| **Preparedness and response planning** | |  | |  |
| --- | --- | --- | --- | --- |
| **1** | **Availability of disaster preparedness and Response WRITTEN plan** | **Completed** | **In progress** | **Not started** |
|  | **First edition** |  |  |  |
|  | **Last update** |  |  |  |
| **2.** | **Content of the plan** |  |  |  |
|  | **1. Surgical emergency management** | **Completed** | **In progress** | **Not started** |
|  | **2. Medical emergency management** | **Completed** | **In progress** | **Not started** |
| **3.** | **Is it evaluated by a drill/simulation?** | **Yes** | **Planning** | **No** |
| **Regarding the Focal point** | |  | |  |
| **1.** | **Is Disaster focal point appointed?** | **Yes** | **Planning** | **No** |
| **2.** | **Permanent or temporary appointment** | **Permanent** | | **Temporary** |
| **3.** | **Working experience in the health sector** |  |  |  |
| **4.** | **Working experience in the institution** |  |  |  |
| **5.** | **Experience in managing real time disasters** |  | |  |
|  | **1. Medical emergencies** | **Yes** | | **No** |
|  | **2. Surgical emergencies** | **Yes** | | **No** |
| **6.** | **Is he/she has undergone disaster training** | **Yes** | **Planning** | **No** |

**ICS ASSESSMENT: Ranking system**

**No capacity at all 0**

**Clear need for improvement 1 (<25%)**

**Basic level of capacity in place 2 (25 – 49%)**

**Moderate level of capacity in place 3 (50 – 74%)**

**High level of capacity in place 4 (75%≤)**

|  | **Category /Action** | | | | | | | | | | | | | **Completed** | **In progress** | **Not started** |
| --- | --- | --- | --- | --- | --- | --- | --- | --- | --- | --- | --- | --- | --- | --- | --- | --- |
| **1** | **Command** | 0 | 1 | | 2 | | 3 | | 4 | |  | | | | | |
| 100 | Appointing an Incident commander? | | | | | | | | | | | | |  |  |  |
|  | Functional positions assigned as needed? | | | | | | | | | | | | |  |  |  |
|  | Command centre opened? | | | | | | | | | | | | |  |  |  |
|  | Notification system? | | | | | | | | | | | | |  |  |  |
| **2** |  | | | | | | | | | | | | | | | |
|  | **Control** | 0 | 1 | | 2 | | 3 | | 4 | |  | | | | | |
| 100 | Facility and staff safety assured? | | | | | | | | | | | | |  |  |  |
|  | Situational assessment? | | | | | | | | | | | | |  |  |  |
|  | Departmental implementation of effective initial response? | | | | | | | | | | | | |  |  |  |
|  | Incident action planning for next operational period planned? | | | | | | | | | | | | |  |  |  |
| **3** | **Communication** | | | 0 | | 1 | | 2 | | 3 | | 4 |  | | | |
| 125 | Activation of appropriate groups and call backs? | | | | | | | | | | | | |  |  |  |
|  | Appointing a Public information officer? | | | | | | | | | | | | |  |  |  |
|  | Release of general employee information (hotline, other) | | | | | | | | | | | | |  |  |  |
|  | Drafting of initial media messages and briefing schedules? (Spokesperson/s identified?) | | | | | | | | | | | | |  |  |  |
|  | Notification of event and situation for external partners? | | | | | | | | | | | | |  |  |  |
|  | “Media monitor” appointed? | | | | | | | | | | | | |  |  |  |
| **4** | \| 0 \| 1 \| 2 \| 3 \| 4 \| \| --- \| --- \| --- \| --- \| --- \|     **Coordination** | | | | | | | | | | | | | | | |
| 100 | Internal departmental needs assessments and reporting to command centre? | | | | | | | | | | | | |  |  |  |
|  | Notification of partner hospitals (External agency) and liaison established? | | | | | | | | | | | | |  |  |  |

| **5** |  | | | | | | | | | |
| --- | --- | --- | --- | --- | --- | --- | --- | --- | --- | --- |
|  | **Staff** | 0 | 1 | 2 | 3 | 4 |  | | | |
| 150 | Staff staging (labour pool) established? | | | | | | |  |  |  |
|  | Identification of additional staff capacity? (mobilization) | | | | | | |  |  |  |
|  | Internal or external source or strategy identified? | | | | | | |  |  |  |
|  | Staff capabilities identified (paediatrics, HDU, ICU etc.)? | | | | | | |  |  |  |
|  | Internal or external source or strategy identified? | | | | | | |  |  |  |
|  | Staff check-in required? | | | | | | |  |  |  |
|  | Staff orientation, mentoring, credentialing required for external staff? | | | | | | |  |  |  |
| **6** |  | | | | | | | | | |
|  | **Stuff** | 0 | 1 | 2 | 3 | 4 |  | | | |
| 75 | Resource report from pharmacy, central supply, lab inpatient, ED requested and received? | | | | | | |  |  |  |
|  | Anticipated shortfalls based on event? | | | | | | |  |  |  |
|  | Stakeholders identified? | | | | | | |  |  |  |
| **7** | \| 0 \| 1 \| 2 \| 3 \| 4 \| \| --- \| --- \| --- \| --- \| --- \|   **Space** | | | | | | | | | |
| 200 | Additional triage areas identified? | | | | | | |  |  |  |
|  | Additional emergency department space identified? (for p1) | | | | | | |  |  |  |
|  | Additional critical care space identified? | | | | | | |  |  |  |
|  | Additional medical or surge space identified? | | | | | | |  |  |  |
|  | Patient holding area identified. (for p2 and p3) | | | | | | |  |  |  |
|  | Separate family and media areas designated? | | | | | | |  |  |  |
|  | Adequacy of the Space is categorized? | | | | | | |  |  |  |
|  | Identification of the requires transfers or alternate care site— liaison with partner agencies and hospitals | | | | | | |  |  |  |
| **8** |  | | | | | | | | | |
|  | **Special** | 0 | 1 | 2 | 3 | 4 |  | | | |
| 125 | Identification of security risk to facility? | | | | | | |  |  |  |
|  | Identification of specific communication or media? | | | | | | |  |  |  |
|  | Identification of specific population or cultural needs? | | | | | | |  |  |  |
|  | Identification of demands for Illness generates special resource? | | | | | | |  |  |  |
|  | Identification of Technical expert(s) ? | | | | | | |  |  |  |
| **9** |  | | | | | | | | | |
|  | **Tracking** | 0 | 1 | 2 | 3 | 4 |  | | | |
| 100 | Tagging or tracking of all incident patients? | | | | | | |  |  |  |
|  | Designated person to coordinate patient lists? | | | | | | |  |  |  |
| **10** | \| 0 \| 1 \| 2 \| 3 \| 4 \| \| --- \| --- \| --- \| --- \| --- \|   **Triage** | | | | | | | | | |
| 125 | Adequate personnel and supplies in triage locations? | | | | | | |  |  |  |
|  | Establishment of secondary triage (to OR, CT)? | | | | | | |  |  |  |
|  | Are systematic changes to the standard of care needed to prevent degradation of all services? | | | | | | |  |  |  |
| **11** | \| 0 \| 1 \| 2 \| 3 \| 4 \| \| --- \| --- \| --- \| --- \| --- \|   **Treatment** | | | | | | | | | |
| 125 | Necessity of the Transfers and arrangement of transport? | | | | | | |  |  |  |
|  | Ability to provide definitive care or damage control interventions on at this time (involves decisions among ED, radiology (Xrays, USS), critical care)? | | | | | | | l |  |  |
|  | Are systematic changes to patient care/staffing required to meet demand? (If so, change documentation, staffing, service lines to reflect best possible care) | | | | | | |  |  |  |
| **12** | \| 0 \| 1 \| 2 \| 3 \| 4 \| \| --- \| --- \| --- \| --- \| --- \|   **Transportation** | | | | | | | | | |
| 125 | Identification of Staging and receiving area(s)? | | | | | | |  |  |  |
|  | Identification of Adequate external capacity or capability? | | | | | | |  |  |  |
|  | Identification of Adequate internal capacity (patient movement)? | | | | | | |  |  |  |
|  | Maintaining medical records and belongings accounted for an external transfer? | | | | | | |  |  |  |
|  | Identification of Traffic controls or traffic plans? | | | | | | |  |  |  |
